# Supplementary material for: The causal relationship between the human gut microbiota and pyogenic arthritis: a Mendelian randomization study
Source: Front Cell Infect Microbiol. 2024 Nov 26;14:1452480. doi: 10.3389/fcimb.2024.1452480 (PMC11629706; doi:10.3389/fcimb.2024.1452480)
Supplement: Supplementary file 1 [file DataSheet1.zip › mendelian test/ebi-a-GCST90027642.csv_forest.pdf]

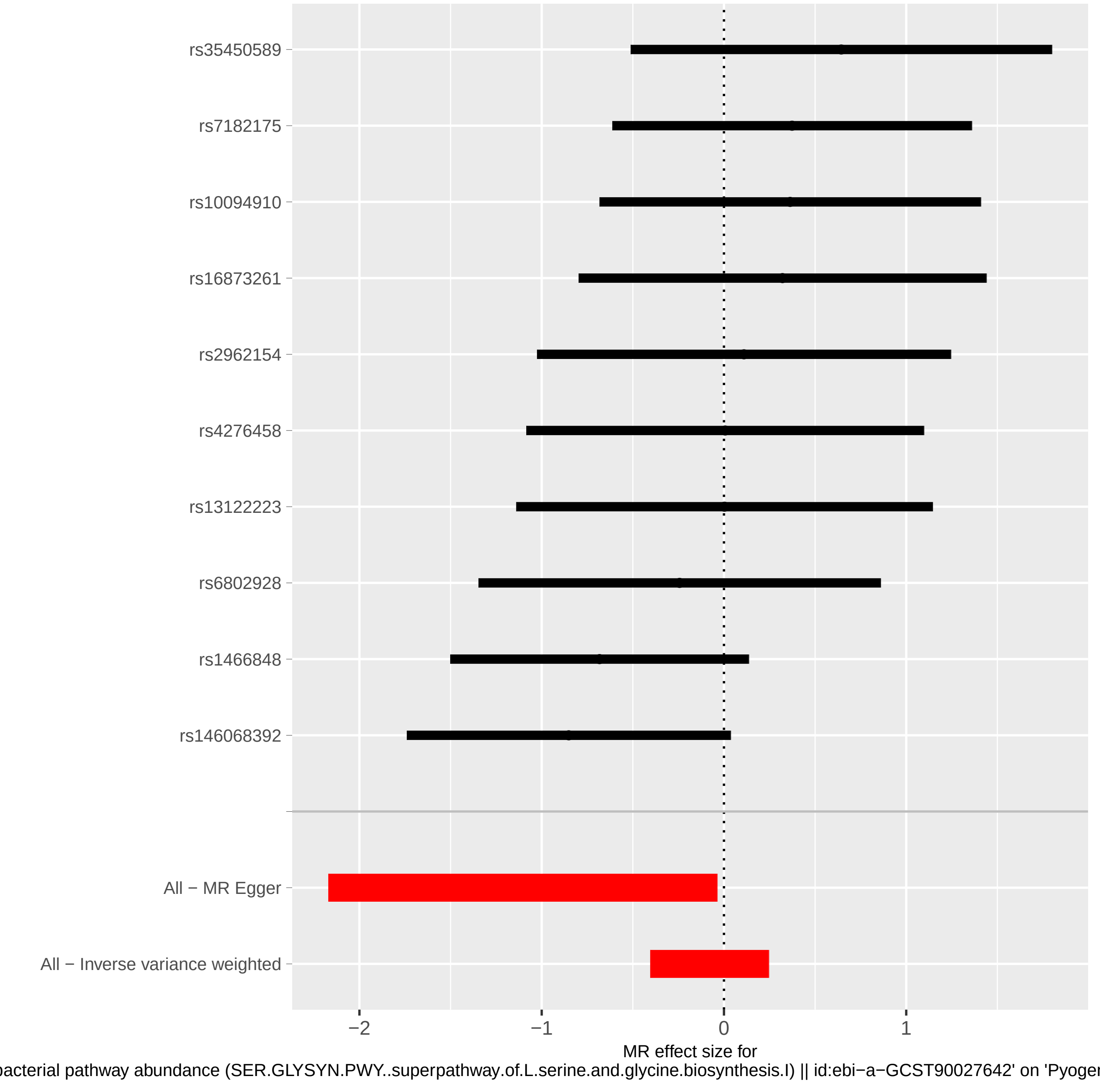

rs35450589
rs7182175
rs10094910
rs16873261
rs2962154
rs4276458
rs13122223
rs6802928
rs1466848
rs146068392
All − MR Egger
All − Inverse variance weighted
−2
−1
0
1
MR effect size for
bacterial pathway abundance (SER.GLYSYN.PWY..superpathway.of.L.serine.and.glycine.biosynthesis.I) || id:ebi−a−GCST90027642' on 'Pyoger
